# Mental Health Professionals’ Experiences of Adapting Mental Health Interventions for Autistic Adults: A Systematic Review and Thematic Synthesis

**DOI:** 10.1007/s10803-023-06006-6

**Published:** 2023-05-14

**Authors:** Laura Moore, Fionnuala Larkin, Sarah Foley

**Affiliations:** School of Applied Psychology, University College Cork, Cork, Ireland

**Keywords:** Adaptations, Mental health interventions, Autistic adults, Thematic synthesis, Autism

## Abstract

Autistic adults experience high rates of metal health difficulties and face significant barriers to accessing appropriate mental health care. Empirical research and recent professional guidelines emphasise the importance of modifying standard mental health interventions to best meet the needs of autistic adults. This systematic review explored mental health professionals’ experiences of adapting mental health interventions for autistic adults. A systematic search was conducted on CINAHL, PsychINFO, PubMed, Scopus, and Web of Science in July 2022. The findings from 13 identified studies were synthesised using thematic synthesis. Three major analytical themes were generated, the unique experience of adapting interventions for autistic clients, factors which facilitate successful adaptations, and challenges to adapting interventions. Each theme contained a number of subsequent sub-themes. Professionals view the process of adapting interventions to be a highly individualised process. A range of personal traits, professional experiences, and systemic, service-based issues were identified in facilitating or challenging this individualised process. Further research regarding adaptations with different intervention models and increased supportive resources are required to enable professionals to successfully adapt interventions for autistic adult clients.

Autism is a neurodevelopmental condition which is characterized by difficulties in social interaction and social communication, as well as restricted or repetitive patterns in behaviour, interests, and activities (American Psychiatric Association, 2013). Until the last decade, autism research has focused primarily on improving outcomes for autistic children and their families (Howlin, 2021). Increasingly, however, adults are accessing and receiving autism diagnoses (Pellicano et al., 2022). Later diagnosis in adulthood is associated with poorer mental health and an overall reduced quality of life (Atherton et al.,^ 2022^). A significant proportion of autistic people^1^ will experience mental health difficulties at some point throughout their lives (Hollocks et al., 2019; Lai et al., 2019; Lever & Geurts, 2016). A recent umbrella review of systematic reviews and meta-analyses found a prevalence rate of 54.8% for at least one psychiatric disorder amongst autistic adults, with anxiety and mood disorders being the most common mental health presentations (Hossain et al., 2020).

The same umbrella review by Hossain et al. (2020) found that autistic adults experienced elevated levels of suicidality. Cassidy et al. (2018) examined risk markers associated with the increased risk of suicidality and found that risk markers shared with the general population, such as non-suicidal self-injury, employment, and mental health, were significantly more prevalent for autistic adults. This study also found that an autism diagnosis was, in itself, an independent risk factor for suicidality, and also identified unique risk markers specifically related to autism, such as the harmful impact of camouflaging autistic traits and having unmet support needs across a number of areas including mental health care, employment, housing, and education (Cassidy et al., 2018). Evidently, there are unique factors related to autism which need to be considered in the assessment and treatment of comorbid mental health difficulties experienced by autistic adults.

The high prevalence rates of mental health difficulties and the associated significant consequences highlight a need for specific intervention or services to improve outcomes for autistic adults. However, autistic adults regularly report barriers to accessing appropriate mental health care (Brede et al., 2022; Camm-Crosbie et al., 2019; Crane et al., 2019; Petty et al., 2021). There are a number of factors which likely contribute to this difficulty. Research from the UK by Crane et al. (2019) found that young autistic adults reported poor management of transitions between youth and adult services and lengthy waiting times for mental health intervention. Autistic adults with comorbid mental health difficulties, without intellectual disability, also regularly fall between the gap of mental health services and specialist autism services (Camm-Crosbie et al., 2019). This lack of specialist care often means autistic adults are treated in generic mental health services. Young adult participants in Crane et al.’s (2019) study highlighted that they did not benefit from accessing treatment designed for a neurotypical population. This may be due to uncertainty around referral routes, difficulties with phone-based communication, sensory sensitivities in new environments, as well as the organisational demands of arranging appointments and completing homework tasks (Brede et al., 2022; Camm-Crosbie et al., 2019; Crane et al., 2019; Petty et al., 2021). One systematic review of 12 studies found that a lack of therapist knowledge of autism or therapists’ unwillingness to tailor their approaches to intervention were the most commonly reported barriers to accessing psychological treatments for autistic individuals (Adams & Young, 2021). There may even be negative consequences for autistic people in accessing and receiving mental health support which is not specifically targeted to their individual needs. A systematic review by Brede et al. (2022) highlighted that the mental health support currently available can result in autistic adults feeling misunderstood or dismissed, due to their use of camouflaging, which can have a negative impact on wellbeing, relationships, and the likelihood of them engaging in future mental health supports.

These system level and clinician level barriers could be further compounded by client level factors associated with autism. For example, the social communication challenges associated with autism may make it difficult for an autistic client to accurately communicate the extent of their distress within a mental health setting (Cooper et al., 2018). The prevalence of alexithymia amongst autistic individuals (Kinnaird et al., 2019) may impact on an individuals’ ability to access standard emotion focused therapies. NICE guidelines highlight that staff delivering interventions for autistic adults should have “an understanding of the core features of autism and their possible impact on the treatment of coexisting mental disorders” (National Institute for Health and Care Excellence (NICE), 2021). However, quantitative research with mental health practitioners and medical professionals highlights limited confidence, training, and comfort in providing adapted care for autistic individuals (Brookman-Frazee et al., 2012; Cooper et al., 2018).

Although there is a lack of appropriate service provision and a number of barriers to accessing services, there is a growing body of evidence that common mental health interventions (MHI) can be used effectively for autistic adults. Research demonstrates modest effect sizes for the efficacy of CBT and mindfulness in the treatment of anxiety and depression (Howlin & Magiati, 2017; Sizoo & Kuiper, 2017; White et al., 2018). A recent systematic review by Linden et al. (2022) of over 71 RCTs found that mindfulness-based interventions had a small effect on anxiety for autistic adults and a medium effect on depression. Adaptations to standard MHI are frequently used in practice and are viewed as important by autistic people (Brice et al., 2021). There is limited research specifically focused on the impact of adaptations on MHI for autistic people. However, the same systematic review as above found that compared to non-adapted, self-directed CBT, self-directed CBT which had been adapted was more effective in reducing depression and anxiety symptoms for autistic adults (Linden et al., 2022).

In line with these findings, professional guidelines for treatment and intervention for mental health difficulties with autistic adults recommend adaptations and the individual tailoring of therapeutic support (National Institute for Health and Care Excellence (NICE), 2021). These guidelines outline that adaptations should include:

- “A more concrete and structured approach with a greater use of written and visual information (which may include worksheets, thought bubbles, images and ‘tool boxes’)
- Placing greater emphasis on changing behaviour, rather than cognitions, and using the behaviour as the starting point for intervention
- Making rules explicit and explaining their context
- Using plain English and avoiding excessive use of metaphor, ambiguity and hypothetical situations
- Involving a family member, partner, carer or professional (if the autistic person agrees) to support the implementation of an intervention
- Maintaining the person’s attention by offering regular breaks and incorporating their special interests into therapy if possible (such as using computers to present information)” (NICE, 2021, p.32).

There have been some efforts at characterizing adaptations for interventions used with young people on the autism spectrum. A review of 83 articles by Dickson et al. (2021) found the most common adaptations used when tailoring CBT interventions for autistic youth with mental health difficulties were increased involvement of parents and autism specific strategies to increase engagement such as use of visuals or special interests. Changes to the content and structure were also frequently reported in order to introduce less material and reducing the length of intervention. Research which examined the use of psychosocial treatments targeting depression and anxiety symptoms in autistic adolescents and adults identified that adaptations to mindfulness interventions included eliminating poetry or metaphors and changing the length of meditations (White et al., 2018). Adaptations to CBT interventions for the same population included increased parent involvement, increased use of structure and visuals, concrete examples, and language, and increased psychoeducation on emotions (White et al., 2018). Such adaptations appear appropriate and important, yet it is unclear how clinicians make decisions about how to adapt MHI for autistic adults or what impact these adaptations have on their clinical practice.

From the perspective of the autistic community, a survey of 537 autistic adults identified a number of adjustments that were deemed to be important in improving the accessibility and acceptability of mental health services (Brice et al., 2021). The adjustments identified were: adjustments to the sensory environment, such as noise and light levels, adjustments to the service context, such as length of appointments or additional information prior to appointments, and adjustments to clinician communication, such as formality of approach or clinicians understanding of autism. These are somewhat different to the adaptations recommended by NICE (2021), but reinforce the importance of modifications in improving, not just the efficacy of MHI, but also the accessibility and acceptability of interventions for autistic adults. Notably, although deemed important, autistic mental health service users reported that these adjustments were often not available in their experience of mental health care (Brice et al., 2021). Therefore, the current study aims to understand how clinicians apply the available guidelines to their practice and what factors facilitate or impede clinicians in adapting MHI for autistic adults.

## The Present Study

Based on the available research and guidelines there appears to be a consensus that adaptations are important in improving the accessibility, acceptability, and efficacy of MHI for autistic adults. However, previous research with autistic mental health service users highlights that, in practice, adaptations are frequently not available, and this is viewed as a barrier to accessing mental health support (Adams & Young, 2021; Brice et al., 2021). Given that the onus is on professionals to appropriately identify, incorporate, and develop such adaptations it is important to understand professionals’ perspectives on this matter. Therefore, the current systematic review will synthesise qualitative research regarding how mental health professionals experience adapting MHI for use with autistic adults. The aim of the current review is to understand clinicians experience of adapting MHI for autistic adults and to specifically respond to the following research questions:

- How do clinicians experience adapting MHI for autistic adults?
- What factors facilitate professionals in adapting MHI for autistic adults?
- What challenges do professionals experience adapting MHI for autistic adults?

## Methodology

This systematic review was conducted and reported with reference to PRISMA guidelines (Page et al., 2021) and in line with the ENTREQ guidelines (Tong et al., 2012). ENTREQ consists of 21 items which cover the five main areas of a qualitative systematic review: introduction, methodology, results, literature search and selection, appraisal, and data synthesis. The systematic review protocol was registered on PROSPERO on 15/06/22, registration number: CRD42022340037.

### Research Question

The ‘PICo’ framework (Lockwood et al., 2015) for developing qualitative research questions for systematic reviews was used to formulate the research question and search strategy. In line with this framework, the *population* referred to mental health professionals including psychologists, counsellors, psychotherapists, mental health nurses, occupational therapists, social workers, and psychiatrists, the *interest* referred to the experience of adapting MHI, and the *context* was for autistic people.

### Inclusion and Exclusion Criteria

Studies were included if they were qualitative and focused on the experiences of mental health professionals adapting MHI for autistic adults. Qualitative components of mixed method studies were included if qualitative results were presented as distinct from the quantitative findings. Mental health professionals could include, but was not limited to, psychologists, psychiatrists, social workers, occupational therapists, and mental health nurses.

Papers were excluded if they were primarily opinion articles, not empirical research. Studies were excluded if they explored only professionals’ experiences of adapting MHI for autistic children. At the exploratory search stage, it became clear that adaptations used with adults varied sufficiently from those used with children and merited a focused qualitative synthesis. Quantitative studies were excluded as the research question is focused on clinicians’ subjective experiences. Studies not available in English were also excluded.

### Search Strategy

Electronic pre-planned searches were conducted across five databases: CINAHL, PsychINFO, PubMed, Scopus, and Web of Science in July 2022. The first five pages of Google Scholar and the reference lists of included articles were screened to identify any further relevant articles. An additional database search was conducted in December 2022 to identify any more recent publications. Search terms were adapted to individual databases (Table 1). The search strategy was assessed by the primary researcher using the Peer Review of Electronic Search Strategies (PRESS) checklist and was found to be in keeping with these guidelines (McGowan et al., 2016).

**Table 1:** Example search strategy for web of science

Search number	Search string
#1	autism spectrum disorder OR autism OR ASD OR Asperger*
#2	Psychological therap* OR psychological intervention* OR therapy OR CBT OR DBT OR EMDR OR anxiet* or depress* OR mental health OR self harm OR suicid* OR mental health treatment* OR mental health intervention
#3	adapt* OR modif* OR adjust* OR alter*
#4	Mental health profess* OR psychologist* OR counsellor* OR psychotherapist* OR nurse* OR occupational therapist* OR psychiatrist* OR social worker
#5	#1 AND #2 AND #3 AND #4

### Screening Process

**Fig. 1:**
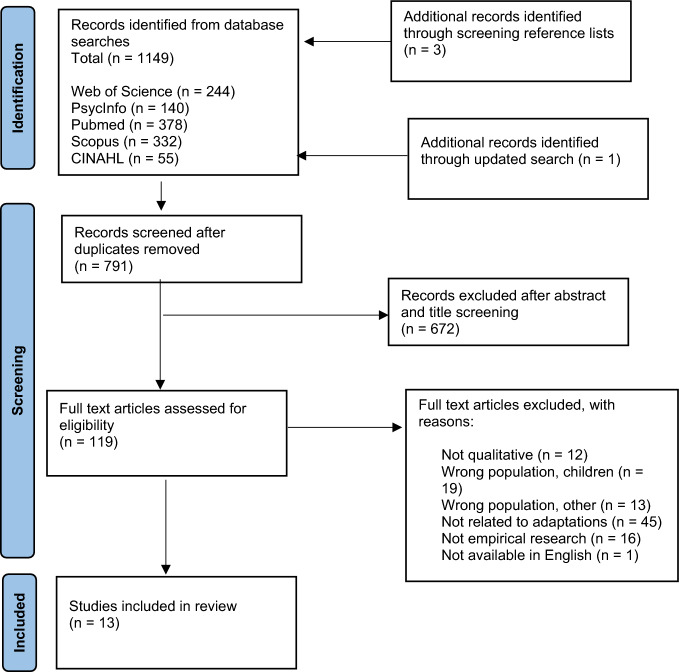
PRISMA flow diagram

Once database searches were complete and all duplicates were removed using Rayyan, the primary author screened all titles and abstracts. The second author screened 20% of the titles and abstracts and any conflicts (*n* = 11) were resolved through discussion. The primary author then screened all remaining full texts for relevance and a third author screened 20% of these full texts. Again, conflict (*n* = 1) was resolved through discussion, until it was agreed that 13 papers met the criteria for inclusion in this review (Fig. 1).

### Data Extraction

Demographic and methodological information was extracted from each study using a pre-planned data extraction table. The results section of each of the primary studies was extracted into N-Vivo to facilitate data analysis.

### Data Synthesis

An inductive approach was used to develop themes and subthemes from the results sections of included studies. The extracted data was synthesised using thematic synthesis. It has been suggested that thematic synthesis can be particularly appropriate for descriptive syntheses targeted at informing policy and clinical practice (Thomas & Harden, 2008). Thematic synthesis involves three steps; line by line coding of the extracted data, developing descriptive themes, and generating analytical themes (Thomas & Harden, 2008). The results section of each included study was extracted to NVivo. In NVivo, a code was created for each line of text, with subsequent data either being assigned to a pre-existing code or given a new code where necessary. This process developed a set of 113 codes which enabled ideas within studies to be translated and synthesised across articles.

These codes were then examined for similarities and differences and grouped into descriptive themes. Groups of codes were given new codes in order to capture their meaning related to the review question. This enabled “going beyond” the content of the primary studies and the development of analytical themes which introduced new concepts and ideas, specific to the research question of the current systematic review, that had not been visible in the primary studies alone.

## Results

### Study Selection

A total of 791 unique studies were identified. After the screening of abstracts, 119 papers remained, and these were assessed for eligibility against the inclusion criteria. This resulted in a further 106 papers being excluded for the reasons outlined in Fig. 1. Thirteen papers were included in the review. The demographic and methodological characteristics of these studies are outlined in Table 2. In total, there were 197 participants included across the 13 studies. The most frequently sampled profession was psychology, however, counsellors (Hume, 2022; Mitran, 2022), mental health nurses (Cooper et al., 2018; Kinnaird et al., 2017; Spain et al., 2017), social workers (Cooper et al., 2018), occupational therapists (Cooper et al., 2018; Kinnaird et al., 2017; Petty et al., 2021, 2022) and psychiatrists (Heijnen-Kohl et al., 2022; Spain et al., 2017) were also represented. The majority of the studies occurred in the UK (Ainsworth et al., 2020; Cooper et al., 2018; Kinnaird et al., 2017; Petty et al., 2021, 2022; Russell et al., 2019; Siddell, 2022; Spain et al., 2017), two studies were from the USA (Maddox et al., 2020; Mitran, 2022), and there was one study each from New Zealand (Hume, 2022) and the Netherlands (Heijnen-Kohl et al., 2022).

**Table 2:** Demographic and methodological information

Reference (year), location	Study aims	Qualitative data collection method	Data analysis	Participants professions (N)	Intervention	Characteristics of intervention recipients
Siddell (2022), UK	To explore the views of clinical psychologists working therapeutically with people with both ASD and ID, particularly in relation to accessibility and effectiveness of psychological interventions	Semi-structured interviews	Thematic analysis	Clinical psychologists (7)	Various models of therapy including -psychodynamic, cognitive analytic therapy, person-centred, CBT and systemic	Adults with ID across South and West Yorkshire in the UK
Ainsworth et al. (2020), UK	To explore practitioners’ experience of anxiety in autistic adults	Semi-structured interviews	Thematic analysis	Clinical psychologists (6) Consultant clinical psychologist (1) Nurse practitioner (1)	Psychological therapy	Adults with ASD using an NHS adult mental health service
Mitran (2022), USA	To explore the experiences of mental health providers when working with neurodiverse clients	Semi-structured interviews	Discourse analysis	Licensed counsellors (5)	Interventions in either a group or private practice setting with neurodiverse clients including a narrative model, EMDR, social thinking approach, human development model, curiosity, role reversal, DBT, goal oriented, proper screening, de-escalation techniques, and script responses	Neurodiverse adult counselling clients
Heijnen-Kohl et al. (2022), Netherlands	To describe what professional experts in clinical practice currently consider specific diagnostic and/or therapeutic aspects of autistic older adults	Delphi method	Consensus analysis	Geriatric psychiatrists (2) Clinical psychologists (2) Clinical neuropsychologists (2) Mental health care psychologists (5)	Not specified	Autistic older adults
Russell et al. (2019), UK	To understand guided self-help coaches’ views of a trial on the use of guided self-help for depression in autistic adults	Semi-structured interviews	Thematic analysis	Assistant psychologists (4) Trainee clinical psychologist (1)	10 sessions of guided self-help which was based on the principles of CBT and included behavioural activation	Autistic adults with depression
Spain et al. (2017), UK	To ascertain professional perspectives about social anxiety (SA) in ASD, and to establish how, if at all, clinicians and researchers adapt their practice when working with this clinical population	Focus groups	Thematic analysis	Adult consultant psychiatrists (5) Child and adolescent psychiatrists (2) Speciality doctors (2) Clinical psychologists (2) Trainee clinical psychologists (3) CBT therapist (1) Nurse specialist (1) Researchers (6)	Not specified	Autistic adults with social anxiety
Hume (2022), New Zealand	To take a first step toward providing more robust guidelines for relationship building between autistic adults and their healthcare professionals	Semi-structured interviews	Creative analytic practice	Mental health counsellors (2) Clinical psychologist (1)	Counselling and psychotherapy	Autistic adults attending support services
Cooper et al. (2018), UK	To survey a sample of UK based psychological therapists, to investigate their current knowledge and past experience of working within a cognitive behavioural framework with autistic people	Open ended questions with free text box	Quantitative content analysis	CBT therapists (50) Nursing (46%) Other core training (30%) Clinical psychologists (18%) Occupational therapy (2%) Social work (2%) No response (2%)	CBT	Autistic adults attending CBT for anxiety, depression, anger, and/or substance abuse
Maddox et al., (2020), USA	To identify barriers and facilitators to providing quality mental health care for autistic adults	Focus groups and individual interviews	Thematic analysis	Community mental health clinicians (44)	Outpatient psychotherapy	Autistic adults
Babb et al. (2021), UK	To triangulate a more rounded understanding of autistic women’s experiences of eating disorder services	Individual interviews	Thematic analysis	Clinical psychologists (6) Psychiatrists (4) Counselling psychologist (1)	Clinical work in eating disorder services	Autistic adult women with eating disorders
Kinnaird et al. (2017), UK	To understand how clinicians approach treating comorbid anorexia and ASD, and how they adapt their typical therapeutic techniques for these patients	Semi-structured interviews	Thematic analysis	9 clinicians including nurse therapists, CBT therapists, cognitive analytical therapist, psychotherapist, dietitian, occupational therapist	Not specified	Autistic adults with an ED attending outpatient eating disorder team
Petty et al. (2021), UK	This study sought the agreed and prioritised recommendations for adapting therapy services for autistic clients by utilising the knowledge of a specialist autism service in the UK	Freelisting–qualitative ethnographic interviewing	Freelisting analysis	Clinical psychologists (5), assistant psychologists (2), speech and language therapists (2), occupational therapist (1), senior psychotherapist (1), medical secretary (1), senior administrator (1), chief officer of clinical services (1) customer relations manager (1)	All worked in a specialist service which provided diagnostic, post-diagnostic and therapeutic services for children, young people and adults	Not specified
Petty et al. (2022), UK	The aim of this study was to ask specialist clinicians about their individual and service-level adaptations to practice for autistic adults	Semi-structured interviews	Thematic analysis	Clinical psychologists (4) Assistant psychologists (2) Occupational therapist (1)	All clinicians delivering therapeutic interventions for autistic adults within a specialist autism services	Autistic adults attending specialist autism service

### Quality Appraisal

All studies that met the inclusion criteria were subject to quality appraisal using the Critical Skills Appraisal Programme (CASP) qualitative checklist (CASP, 2018). The CASP is a widely used 10-item checklist. Nine of the items relate to the validity and presentation of results and these items are examined in Table 3 (Appendix 1). The studies included in this review appeared to be of a relatively high standard. However, there are limitations in the studies related to the use of convenience sampling, limited sample sizes, and the varying levels of autism specific experience held by study participants. These limitations suggest that some caution should be applied when considering the generalizability of results within the included studies. The final question of the CASP considers how valuable the research is. The majority of articles included in the current review attempt to add value to the field by making recommendations for practice or future research. No studies were excluded on the basis of quality assessment.

### Results of Thematic Synthesis

Three overarching categories, consisting of 10 analytical themes, were established as a result of thematic synthesis:

- *The Unique Experience of Adapting Interventions for Autistic Adults,* which included different expectations, individual nature of adaptations, differences in communication, and attitudes towards the work.
- *Factors which Facilitate Successful Adaptations of Interventions,* which included flexibility in practice, prior experience with autism, and positive impact of other relationships.
- *Challenges in Successfully Adapting Interventions,* which included complexity of autism, limited resources, and an inflexible system.

### Unique Experience of Delivering Adapted Interventions

#### Different Expectations

Within the reviewed studies, professionals described adjusting their expectations towards MHI for autistic clients (Petty et al., 2021; Russell et al., 2019; Siddell, 2022; Spain et al., 2017); “I think that this kind of, sit down 1:1 in a room, that’s a classic notion of therapy and sometimes I think that’s very hard…” (Siddell, 2022, p. 6). Therefore, professionals described adjusting MHI by incorporating preparatory work which often focused on emotions (Ainsworth et al., 2020; Petty et al., 2021; Spain et al., 2017). This was captured by one professional: “I think there’s pre-therapeutic work to do just getting people to trust, and emotional recognition, labelling thoughts, understanding how it all links together.” (Spain et al., 2017). Interventions often moved at a slower pace to allow clients to gradually build familiarity with thinking in this way (Cooper et al., 2018; Petty et al., 2021, 2022; Russell et al., 2019; Siddell, 2022; Spain et al., 2017).

Many of the studies also described how expectations around outcomes are different when adapting MHI for autistic clients. For example, clients may report increased symptoms, but the intervention may have been successful in enabling the client to better describe or identify symptoms (Ainsworth et al., 2020; Mitran, 2022; Siddell, 2022; Spain et al., 2017). Some participants also highlighted how difficulty generalizing outside of session and rigidity, associated with autism, can also contribute to this difficulty with measuring progress and how this changes the professionals’ role (Ainsworth et al., 2020; Babb et al., 2021; Russell et al., 2019; Spain et al., 2017). For example, Spain et al., (2017) detailed differences in expectations from clients: “What a change looks like in their mind, it might be ‘I have to be 100% better and nothing’s better until I’ve reached that point’ but actually our whole job is pointing out the shades of grey …..” (Spain et al., 2017, p. 16). Delivering adapted MHI requires professionals to adjust their own outlook regarding the therapeutic process and the evaluation. This finding in relation to expectations, outcomes, and evaluation of MHI for autistic adults is not considered within the available guidelines and recommendations for practice.

#### Individual Nature of Adaptations

There was a clear sense that appropriate adaptations are viewed as a key component of effective MHI for this client group. Adaptations were associated with more positive outcomes, more meaningful work, and improved client engagement (Ainsworth et al., 2020; Heijnen-Kohl et al., 2022; Kinnaird et al., 2017; Russell et al., 2019; Siddell, 2022). Clinicians’ personal skills and experiences contributed to the particular modifications they make (Babb et al., 2021; Siddell, 2022). Some viewed a cognitive focus to adaptations as effective and appropriate (Russell et al., 2019; Spain et al., 2017), whereas others modified a CBT approach by focusing more heavily on behavioural components of intervention (Ainsworth et al., 2020; Heijnen-Kohl et al., 2022; Petty et al., 2022). Professionals describe a focus on making adaptations on an ad hoc basis dependent on the particular client in front of them, therefore adaptations can be viewed as “catered interventions to the client rather than any particular defined diagnosis of neurodiversity” (Mitran, 2022, p. 6).

An area which a number of reviewed studies highlighted was the use of special interests in order to tailor interventions and approaches to meet the individual needs of autistic clients (Ainsworth et al., 2020; Kinnaird et al., 2017; Siddell, 2022). One participant described having a “tool kit of interventions that can be customised to a particular patient (Spain et al., 2017, p. 18). There was an overall consensus across studies that the adaptations professionals make to MHI do not take a standard format and instead are highly individualised based on the unique needs of individual clients, which is in line with NICE guidance and research with autistic mental health service users (Brice et al., 2021).

#### Differences in Communication

The majority of studies described how professionals adapt their own language use and communication style as part of adaptations to MHI for autistic adults. One participant reported; “it’s all about putting an autistic lens on and saying, how could I explain this differently?” (Babb et al., 2021, p. 1416). The majority of studies specifically noted adapting language by using clear, concrete, literal language and avoiding metaphors (Ainsworth et al., 2020; Cooper et al., 2018; Heijnen-Kohl et al., 2022; Kinnaird et al., 2017; Mitran, 2022; Petty et al., 2021; Siddell, 2022; Spain et al., 2017). Other communication adaptations included written diaries, communicating by phone or video and increased use of visual aids (Ainsworth et al., 2020; Cooper et al., 2018; Kinnaird et al., 2017; Spain et al., 2017).

Clients’ difficulties communicating their own distress was sometimes described as uniquely challenging (Ainsworth et al., 2020; Cooper et al., 2018; Kinnaird et al., 2017; Siddell, 2022; Spain et al., 2017). Many studies described the process of building a therapeutic relationship and facilitating the clients’ engagement as being difficult (Ainsworth et al., 2020; Babb et al., 2021; Cooper et al., 2018; Kinnaird et al., 2017). A number of studies described how facilitating relationship-building became a core component of working with autistic clients as highlighted by this quote; “This is who I am, and I’m really interested in who you are. It’s okay for you to come here and be you” (Hume, 2022, p. 157). This process required ongoing attention from professionals. Two studies noted how autistic adults’ communication styles could negatively impact professionals’ experience of the therapeutic relationship and could contribute to a wider view of the individual client as difficult or rude (Kinnaird et al., 2017; Russell et al., 2019). This finding reinforces the importance of adaptations to communication as is well documented within the available research. Here, it also highlights the impact of communication-based misunderstandings, in either direction, on the therapeutic relationship.

#### Attitudes Towards Work

Studies reported various attitudes towards the work of adapting MHI for use with autistic clients. Three studies, in particular, highlighted how professionals experience this work as rewarding (Hume, 2022; Mitran, 2022; Siddell, 2022). These professionals described a joy and passion for their work; Hume (2022, p.157) reports: “They all expressed love for working with their autistic clients…” There was also an acknowledgement that providing intervention for autistic clients can be emotionally affecting, sometimes due to an awareness of client’s previous negative interactions with mental health professionals (Hume, 2022).

Other studies highlighted ambivalent attitudes towards adaptations to MHI for autistic adults. Many professionals did not consciously decide to work with autistic clients and highlighted that professional interest in mental health did not necessarily include autism (Babb et al., 2021; Mitran, 2022). Professionals frequently described feeling uncertain and uninformed regarding approaches to adapting interventions for use with autistic clients which often led to fear around this work (Ainsworth et al., 2020; Kinnaird et al., 2017; Maddox et al., 2020). One participant described it as “and that could be because of unfamiliarity. That could be because of fear that you wouldn’t be able to know what to do” (Maddox et al., 2020, p. 9). This theme highlights the uncertainty experienced by professionals regarding the work of adapting MHI. It seems likely that professionals with passion for the area would be more motivated to focus on unique adaptations required by each client. This theme provides novel insights that enhance our understanding of how clinicians, in practice, experience their work adapting MHI for autistic adults.

### Factors Which Facilitate Successful Adaptations of Interventions

#### Flexibility in Practice

Most of the studies highlighted a need for flexibility from professionals when adapting interventions for autistic adult clients. Professionals placed a strong emphasis on thinking outside the box, using creative approaches to intervention work, being open minded about individual clients’ experiences of specific interventions, and being willing to change practicalities around appointments such as length, time, and format (Ainsworth et al., 2020; Kinnaird et al., 2017; Mitran, 2022; Petty et al., 2021, 2022; Russell et al., 2019; Siddell, 2022; Spain et al., 2017). One psychologist noted “As I am talking about this I realise, oh I did that with one person and that other thing with one other person. I think it’s all about thinking ultra-flexibly” (Siddell, 2022, p. 6).Many participants described the importance of taking a trial-and-error approach as there is “no hard and fast rule about which techniques work best” for this client group (Spain et al., 2017, p. 18). Professionals appeared to rely on their clinical intuition when it came to knowing when and which adaptations to use (Ainsworth et al., 2020; Siddell, 2022; Spain et al., 2017). Others emphasised the importance of creativity, resourcefulness, and using initiative in most effectively seeking information and adapting resources and techniques for the individual needs of clients (Ainsworth et al., 2020; Kinnaird et al., 2017; Mitran, 2022). Therefore, a clinician level capacity and willingness to be flexible in their practice appears to be an important facilitator to the appropriate adaptation of MHI for autistic adults, as recommended in available guidelines.

#### Prior Experience with Autism

The extent of professionals’ previous experience and knowledge of working with autistic clients varied across the studies in this review. However, it was frequently noted that the extent of experience held by professionals contributed significantly to their comfort and confidence with making adaptations to suit their autistic clients (Ainsworth et al., 2020; Babb et al., 2021; Kinnaird et al., 2017; Maddox et al., 2020; Mitran, 2022; Siddell, 2022). It was also acknowledged that because this is so individual to each professional, there can be significant discrepancies with the interventions offered within and between services (Ainsworth et al., 2020; Babb et al., 2021). As well as prior experience of working with autistic clients, a number of studies also highlighted the role of formal training or personal experience of autism in facilitating the successful adaptation of interventions (Maddox et al., 2020; Mitran, 2022; Petty et al., 2021; Russell et al., 2019). One study noted “All coaches described how, after receiving the training, they felt knowledgeable, confident and well prepared to deliver the intervention” (Russell et al., 2019, p. 56). This finding highlights the ability to adapt MHI for autistic adults as a skill which can develop with time and experience.

#### Positive Impact of Other Relationships

The majority of studies noted the importance of including important people in the client’s life in interventions. This could assist clients with communication difficulties, support clients in generalizing outside of intervention sessions, and provide valuable collateral information (Ainsworth et al., 2020; Heijnen-Kohl et al., 2022; Kinnaird et al., 2017; Mitran, 2022; Petty et al., 2021; Siddell, 2022; Spain et al., 2017). Two studies highlighted almost a reliance on the involvement of others as a critical component of successful interventions with autistic clients (Ainsworth et al., 2020; Cooper et al., 2018).

A number of studies also highlighted the importance of relationships with other professionals facilitating adapted MHI. The ability to work collaboratively and to offer and receive support was valued by professionals (Heijnen-Kohl et al., 2022; Kinnaird et al., 2017; Petty et al., 2021, 2022; Siddell, 2022). It was clear from a number of studies that professionals felt a strong need for support in the form of training, supervision, and collaborative working in order to alleviate some of the burden of uncertainty and complexity that appears to be a significant component of this work (Ainsworth et al., 2020; Hume, 2022; Kinnaird et al., 2017; Maddox et al., 2020; Mitran, 2022; Russell et al., 2019; Siddell, 2022). “You might speak about some work you’ve done and then a colleague might say ‘‘oh yes I did something similar’’ and that makes you feel less like your off doing things on your own.” (Siddell, 2022, p. 7). From a number of the studies reviewed it was clear that professionals would like collaborative working to be more common (Ainsworth et al., 2020; Kinnaird et al., 2017; Maddox et al., 2020). As noted in one study “It may be the case that increased peer support or knowledge exchange could alleviate some of the difficulties associated with having to rely on resourcefulness in this particular area of practice” (Ainsworth et al., 2020, p. 6). In some ways, the involvement of family members or supportive working relationships with colleagues appeared to alleviate some of the burden on the professional in adapting MHI. This theme reinforces the NICE guidance recommendation of involving others in interventions and draws attention to the importance of support from other professionals in facilitating clinicians to adapt MHI for autistic adults.

### Challenges in Successfully Adapting Interventions

#### Complexity of Autism

Many studies described some difficulty with adapting MHI due to the complexity of distinguishing characteristics of autism from symptoms of mental illness. This concern about diagnostic overshadowing was a particularly prominent feature of studies which focused on interventions with autistic people with comorbid anxiety or eating disorders (Ainsworth et al., 2020; Babb et al., 2021; Kinnaird et al., 2017; Spain et al., 2017). One participant described difficulty “differentiating between anxiety relating to ASD that the client does not want to address and anxiety getting in the way of them living their life in the way they want” (Cooper et al., 2018, p. 48). Professionals experienced this difficulty as “murky and a grey area” (Babb et al., 2021, p. 1413) which complicated their work of appropriately offering adapted interventions. Some professionals viewed clients’ cognitive limitations, such as trouble concentrating, poor retention, or limited understanding, as an aspect of autism which may increase the challenges of adapting and delivering interventions (Cooper et al., 2018; Maddox et al., 2020). Only one study described the specific challenge of the impact of comorbid intellectual disability (Siddell, 2022).

Some studies described how the rigidity, black and white thinking, and focus on routine associated with autism could be challenging (Cooper et al., 2018; Kinnaird et al., 2017; Russell et al., 2019). The following quote illustrates how this can lead clinicians to worry about how the MHI will be perceived “…change makes them anxious so I thought, ‘Gosh, this whole thing is building up to this point where they have to change something’.” (Russell et al., 2019, p. 62). Other studies noted how clients’ sensory sensitivities could add a layer of complexity to interventions, as greater attention needs to be paid to sensory aspects of the environment (Babb et al., 2021; Cooper et al., 2018; Kinnaird et al., 2017; Maddox et al., 2020; Petty et al., 2021, 2022). Although many professionals were enthusiastic about the involving other people in the client’s life in interventions, this can also be uniquely challenging, particularly if there were discrepancies between the client’s goals and the goals of the family member (Ainsworth et al., 2020; Cooper et al., 2018). This finding offers one rationale for why, in practice, clinicians may find it difficult to appropriately adapt MHI.

#### Limited Resources

The majority of studies indicated that professionals’ ability to effectively adapt MHI was limited by a lack of external resources. A lack of training was frequently noted as a limiting factor (Ainsworth et al., 2020; Maddox et al., 2020; Mitran, 2022; Siddell, 2022). One participant described “I don’t see myself helping that person because of my training. It would be a disservice…” (Maddox et al., 2020, p. 8), highlighting the importance of autism specific training in improving clinicians’ abilities and confidence. These studies described how mental health training often does not include a focus on adapting MHI; “… we all recognised that we really don’t have an awful lot of training, and not a lot of training in adaptation for CBT working with this group” (Ainsworth et al., 2020, p. 5). It was also noted that additional training for MDT teams and services would be helpful in facilitating colleagues, with less autism experience, to see the value of professionals approaches to adapting interventions (Kinnaird et al., 2017; Maddox et al., 2020; Siddell, 2022). Two studies identified training delivered by autistic people could be important to increase understanding (Maddox et al., 2020; Siddell, 2022).

A number of studies also emphasise how the limited evidence base regarding adapted MHI impacts professionals’ experiences of carrying out this work (Ainsworth et al., 2020; Heijnen-Kohl et al., 2022; Maddox et al., 2020; Mitran, 2022; Siddell, 2022). One participant described “a lack of research resources to draw upon, despite feeling these would be very helpful” (Ainsworth et al., 2020, p. 6). This lack of evidence-base meant professionals often needed to be more flexible, creative, and put greater time and effort into making adaptations. However, professionals often described that they had limited resources, such as time, materials, and autonomy, which made this challenging (Ainsworth et al., 2020; Cooper et al., 2018; Petty et al., 2021). This theme highlights how a lack of available resources acts as a barrier to clinicians adapting MHI for autistic adults.

#### An Inflexible System

Although professionals value and recognise the importance of a flexible approach, as described within the ‘flexibility in practice’ theme, there was evidence this this was not always valued within the services and systems in which they work. Professionals felt limited by the challenges of “working flexibly in an inflexible system” (Siddell, 2022, p. 6). This was experienced in relation to freedom to make decisions regarding appointment times, session lengths, locations, duration of intervention (Ainsworth et al., 2020; Cooper et al., 2018; Petty et al., 2021). Professionals also experienced inflexibility regarding environmental adaptations which could be useful for their autistic clients (Babb et al., 2021; Petty et al., 2021; Siddell, 2022; Spain et al., 2017). Typical mental health settings were often viewed as inappropriate: “For somewhere as busy as our clinic, having so many people, so many visitors, something like that isn’t designed in a way that’s friendly for ID or ASD really” (Siddell, 2022, p. 4).

Some studies highlighted how professionals experienced the rigidity of service models and policies; “But what’s difficult is when there’s a service model that says work in this way……and you don’t know whether the person’s going to fit in that or not….” (Spain et al., 2017, p. 15). Some studies highlighted how professionals questioned the suitability of services to meet the needs of their autistic clients and how this lack of flexibility contributes to significant variations in the quality of interventions (Maddox et al., 2020; Siddell, 2022; Spain et al., 2017). Some studies also emphasised that this inflexibility led to frustration for professionals (Siddell, 2022; Spain et al., 2017). These findings highlight how wider systemic factors can act as a barrier to clinicians, in practice, adapting MHI for autistic adults by limiting their capacity to provide adjustments and alterations for their clients.

## Discussion

This thematic synthesis explored mental health professionals’ experiences of adapting MHI for use with autistic adults. In keeping with professional guidelines, which recommend “individual tailoring of therapeutic support” (NICE, 2021, p.32), clinicians described the process of adapting MHI as uniquely tailored to each client’s needs. In line with NICE guidance, professionals highlighted their use of adaptations such as communication style, involving others, incorporation of special interests, focus on behaviour, adjustments to intervention pace, length, or duration, as well as environmental adaptations. These findings, regarding the ‘individual nature of adaptations’, ‘differences in communication’, and ‘flexibility in practice’, reinforces the necessity of professional guidelines and offer evidence of how these are applied. Further, the adaptations most frequently noted by professionals within the current systematic review are in keeping with research exploring autistic adults’ views on factors which improve the acceptability of MHI (Brice et al., 2021; Camm-Crosbie et al., 2019; Crane et al., 2019; Horwood et al., 2021; Maddox et al., 2020). This consensus between clinicians and autistic adults offers further support for the central role of appropriate, individualised adaptations to MHI for autistic adults.

NICE guidelines also recommend involving another person to support intervention, where appropriate. Similarly, findings from the current thematic synthesis emphasize the importance of involving supportive others. Results from the current study offer a more nuanced understanding that involvement of others appears to serve a dual purpose. First, it improves accessibility for autistic clients who may struggle within a novel therapeutic setting. Second, this appears to alleviate some of the fear and uncertainty professionals experience in adapting MHI for autistic adults as a result of the complexity of autism and the limited resources available to them. Therefore, supportive input from others facilitates clinicians’ abilities to adapt MHI in practice. Professionals also highlighted how their own capacity and willingness to be flexible in their approach was an essential to successfully adapting interventions for their autistic clients. A systematic review by Adams and Young (2021) highlighted that therapists’ unwillingness to adapt and tailor their approach to intervention was viewed as a significant barrier by autistic adults. Given the significance of this barrier, it is encouraging that many professionals within the reviewed studies described how their training, prior experience of autism, willingness to seek feedback, and ability to adapt practical aspects of appointments enabled the appropriate adaptions required for individual clients’ needs. Again, this highlights the consensus among mental health clinicians and autistic mental health service users regarding the significance of therapist flexibility and willingness to adapt their approach to MHI for autistic adults.

The novel findings from the current thematic synthesis in relation to the challenges to adapting MHI which exist in practice are particularly important. Notably, professionals within the reviewed studies varied in their attitudes towards work adapting MHI for autistic adults. Some professionals felt passionate and emotionally invested in the work. There was evidence of many professionals engaging in creative, resourceful, and reflective practice to provide individually tailored adaptations. Other professionals described feeling more ambivalent, disinterested, unsure, or even fearful of the work. (Ainsworth et al., 2020; Babb et al., 2021; Mitran, 2022). Ambivalence and limited experience likely contribute to a sense of uncertainty and fear in approaching adaptations which could, feasibly, impact the development of a therapeutic alliance, a central component of all MHI (Norcross, 2010). In Hume’s (2022) study, 17 autistic adults were interviewed and emphasized the importance that autistic adult counselling clients place on the therapeutic relationship. The existence of the double empathy problem (Milton, 2012), alongside the communication differences described in the relevant subtheme, explains some of the challenge which exists in forming a therapeutic alliance. This can lead to autistic people being misperceived within mental health services which can maintain mental health difficulties despite treatment (Mitchell et al., 2021). Therefore, it is important that clinicians have a keen understanding of the challenges and adaptations which are required to build therapeutic relationships with autistic adults to facilitate the delivery of MHI.

Professionals’ feelings of fear and uncertainty are further compounded by the limited evidence base and professional guidance. Though there are emerging efforts to introduce professional guidelines on working with mental health and autism (BPS, 2021; PSI, 2022). Such guidelines are likely too recent to have had a significant impact on professionals sampled in the studies reviewed here. Many professionals also felt constrained in their approach to adapting MHI by the limited resources available to them, and a lack of autonomy to alter aspects of their professional practice while working in inflexible systems or services, which are not designed to facilitate the flexibility required by autistic adults. Some of this inflexibility may be informed by the prevalence of the medical model within many mental health services and a tendency towards attempting to fit autistic people into a neurotypical model, rather than a more neurodiverse informed approach (Mitchell et al., 2021).

### Implications for Clinical Practice

Findings from the current systematic review highlight that there is an ongoing need for training for professionals working with autistic people in mental health settings. Importantly, the finding that, in practice, clinicians feel constrained when attempting to work flexibly within ‘an inflexible system’ reinforces the need for ongoing training and education for all involved in the delivery of MHI including the wider multidisciplinary team, management, and policy makers. This would enhance understanding regarding the level of flexibility required in the successful delivery of MHI for autistic adults. Clinicians could then be facilitated and empowered to implement the adaptations deemed important by the findings described here, in available guideline (NICE, 2021), and by autistic people (Brice et al., 2021; Camm-Crosbie et al., 2019). It is crucial that any such training for professionals ensures that autistic people are central to the development, delivery, and evaluation processes given their unique and critical role as autism experts (Fletcher-Watson et al., 2019; Gillespie-Lynch et al., 2017).

Given the unique challenges of the complexity of autism, the limited resources, and the inflexible systems which exist it is understandable that clinicians particularly appreciate collaborative, supportive working with other professionals. Thus, space and time for such reflective and functional working should be prioritised for clinicians whose work routinely involves the adaptation of MHI for autistic adults. This could be facilitated within particular services or across geographic areas. Such emphasis on collaborative sharing would serve to buffer against the fear, uncertainty, and ambivalence described by professionals within the reviewed studies. Consequently, this would facilitate an enhanced understanding and awareness of the unique needs of autistic clients and, ultimately, improve the efficacy of adapted MHI.

With consideration of the highly individual tailoring required, the double empathy problem, and the primary importance of autistic clients feeling understood in adapted MHI (Brice et al., 2021; Mitchell et al., 2021) clinicians, in practice, should routinely seek open discussion and feedback regarding the suitability of any adaptations. At the outset, with each client clinicians should discuss what communication, practical, sensory, or environmental adaptations might be required. During intervention, clinicians should seek feedback from their clients regarding any additional adaptations which might be appropriate. Feedback could be sought through informal discussion or through the use of an evaluative outcome measure. Given the finding from Adam and Young (2021) that therapists’ unwillingness to adapt was the most significant barrier to accessing mental health support for autistic people, this open, collaborative style of working would likely be well received by autistic clients.

### Strengths and Limitations

This systematic review is the first to synthesise qualitative research exploring how clinicians experience adapting MHI for autistic adults. The thematic synthesis extends research with autistic adults by Brice et al. (2021) and illustrates the factors which facilitate and challenge the application of adaptations in practice. The search strategy utilised was robust and quality checked against the PRESS guidelines (McGowan et al., 2016) which provided an additional layer of quality control. All studies, except the study by Heijnen-Kohl et al. (2022), occurred in English speaking countries and all studies have utilised a Western sample of clinicians. Therefore, it is unclear if these findings are relevant cross-culturally. The studies included in this review were heterogeneous, had different aims, utilised different analytical methods, and sampled a broad range of mental health professionals, though psychologists were over-represented. A risk that is present in all qualitative synthesis is that in merging the overall findings from qualitative studies some of the meaning of individual studies is lost (Duden, 2021).

### Suggestions for Future Research

There is a limited, but growing, evidence base regarding the efficacy of MHI for autistic adults and the impact of adaptations to MHI. The majority of the evidence base regarding efficacy is focused on CBT for anxiety and depression (Linden et al., 2022). Frequently, the adaptations are described in vague terms which acts as a barrier to clinicians applying such adaptations in practice. Future studies concerned with the efficacy of MHI for autistic adults would benefit from a defined case study approach of naming and describing the specific adaptations incorporated. As is clear from the papers included in this review, clinicians in practice are utilising a variety of approaches, such as psychodynamic, systemic, and narrative, to their intervention work with autistic people. However, CBT continues to be the main focus of efficacy research. This contributes to clinicians’ uncertainty and frustration with a limited evidence base. Future research in this area should consider a wider variety of treatment models, this would ensure autistic adults are offered a selection of appropriate interventions. Research exploring adaptations to MHI for conditions apart from anxiety and depression, such as obsessive–compulsive disorder or eating disorders, which are also common amongst autistic adults (Hossain et al., 2020), would be important. The studies included in this review sampled a wide variety of mental health providers, such as psychologists, social workers, and occupational therapists, and, frequently, collated the qualitative findings across groups. Future research may benefit from a consideration of the unique perspectives and challenges within each professional discipline. This could highlight particular strengths, areas of weakness, and identify particular roles for different professions in MHI for autistic adults, particularly within multidisciplinary service contexts.

## Conclusion

This systematic review synthesised the findings from 13 qualitative studies exploring mental health professionals’ experiences of adapting MHI for autistic adults. Professionals viewed adaptations which were individualised and based on each clients’ unique needs as central to the successful delivery of adapted MHI. Professionals viewed this work as a unique experience due to the significant need for individual tailoring, an adjustment of their expectation, differences in communication, and the complexity of autism. Although professionals agree with the views of autistic people and professional guidelines regarding the need for a tailored approach to MHI for autistic adults, in practice they felt constrained by the limited availability of resources and the challenges of inflexible systems. Many professionals described uncertainty and fear in their attitudes towards adapting MHI, whereas others described high levels of motivation and creativity. A capacity for flexibility in practice, prior experience with autism, and supportive relationships facilitated professionals in appropriately adapting MHI. Professionals highlighted that increased guidance, evidence, and resources would facilitate their work to appropriately adapt MHI for autistic adults.

## Appendix 1

See Table 3.

**Table 3:** CASP quality assessment table

Study	Siddell (2022)	Ainsworth et al. (2020)	Mitran (2022)	Heijnen-Kohl et al. (2022)	Russell et al. (2019)	Spain et al. (2017)	Hume (2022)	Cooper et al. (2018)	Maddox et al., (2020)	Babb et al. (2021)	Kinnaird et al. (2017)	Petty et al. (2021)	Petty et al. (2022)
Was there a clear statement of the aims of the research?	Y	Y	Y	Y	Y	Y	Y	Y	Y	Y	Y	Y	Y
Is a qualitative methodology appropriate?	Y	Y	Y	Y	Y	Y	Y	Y	Y	Y	Y	Y	Y
Was the research design appropriate to address the aims of the research?	Y	Y	Y	Y	Y	Y	Y	Y	Y	Y	Y	Y	Y
Was the recruitment strategy appropriate to the aims of the research?	Y	Y	Y	Y	Y	Y	CT	Y	Y	Y	Y	Y	Y
Was the data collected in a way that addressed the research issue?	Y	Y	Y	Y	Y	Y	Y	Y	Y	Y	Y	Y	Y
Has the relationship between researcher and participants been adequately considered?	Y	Y	Y	N	Y	Y	N	CT	N	N	Y	N	N
Have ethical issues been taken into consideration?	CT	Y	Y	CT	Y	Y	CT	Y	CT	Y	Y	Y	Y
Was the data analysis sufficiently rigorous?	Y	Y	Y	Y	Y	Y	CT	Y	Y	Y	CT	Y	Y
Is there a clear statement of findings?	Y	Y	Y	Y	Y	Y	Y	Y	Y	Y	Y	Y	Y
How valuable is the research?	V	V	V	V	V	V	V	V	V	V	V	V	V

## Footnotes

1 The terminology used to describe people on the autism spectrum is subject to continued discussion (Botha et al., 2020; Vivanti, 2019). Recent empirical research suggests that autistic individuals tend to prefer identity first language (IFL), “autistic person”, or the term “person on the autism spectrum” over person first language, “person with autism” (PFL) (Bottema-Beutel et al., 2021; Bury et al., 2020; Kenny et al., 2016). Therefore, IFL is used throughout this paper though it is acknowledged that this does not represent the preferences of all individuals on the autism spectrum.

## References

[CR0] *Asterisked papers are the 13 studies which were included in the systematic review.

[CR1] Adams, D., & Young, K. (2021). A systematic review of the perceived barriers and facilitators to accessing psychological treatment for mental health problems in individuals on the autism spectrum. *Review Journal of Aussstism and Developmental Disorders,**8*(4), 436–453. 10.1007/s40489-020-00226-7 10.1007/s40489-020-00226-7

[CR2] *Ainsworth, K., Robertson, A. E., Welsh, H., Day, M., Watt, J., Barry, F., Stanfield, A., & Melville, C. (2020). Anxiety in adults with autism: Perspectives from practitioners. *Research in Autism Spectrum Disorders*. 10.1016/j.rasd.2019.101457 10.1016/j.rasd.2019.101457

[CR3] American Psychiatric Association. (2013). *Diagnostic and statistical manual of mental disorders* (5^th^ ed.). American Psychiatric Publishing, Inc. https://psycnet.apa.org/doi/10.1176/appi.books.9780890425596

[CR53] Atherton, G., Edisbury, E., Piovesan, A., & Cross, L. (2022). Autism Through the Ages: A Mixed Methods Approach to Understanding How Age and Age of Diagnosis Affect Quality of Life. *Journal of Autism and Developmental Disorders*, 52(8), 3639–3654. 10.1007/s10803-021-05235-x 34482523 10.1007/s10803-021-05235-x PMC9296439

[CR4] *Babb, C., Brede, J., Jones, C. R. G., Elliott, M., Zanker, C., Tchanturia, K., Serpell, L., Mandy, W., & Fox, J. R. E. (2021). ‘It’s not that they don’t want to access the support … it’s the impact of the autism’: The experience of eating disorder services from the perspective of autistic women, parents and healthcare professionals. *Autism,**25*(5), 1409–1421. 10.1177/1362361321991257 33588579 10.1177/1362361321991257 PMC8264634

[CR55] Botha, M., Hanlon, J., & Williams, G. L. (2020). Does Language Matter? Identity-First Versus Person-First Language Use in Autism Research: A Response to Vivanti. *Journal of Autism and Developmental Disorders*. 10.1007/s10803-020-04858-w 10.1007/s10803-020-04858-w PMC7817071 33474662

[CR6] Bottema-Beutel, K., Kapp, S. K., Lester, J. N., Sasson, N. J., & Hand, B. N. (2021). Avoiding ableist language: Suggestions for autism researchers. *Autism in Adulthood,**3*(1), 18–29. 10.1089/aut.2020.0014 36601265 10.1089/aut.2020.0014 PMC8992888

[CR7] Brede, J., Cage, E., Trott, J., Palmer, L., Smith, A., Serpell, L., Mandy, W., & Russell, A. (2022). “We have to try to find a way, a clinical bridge”-autistic adults’ experience of accessing and receiving support for mental health difficulties: A systematic review and thematic meta-synthesis. *Clinical Psychology Review*. 10.1016/j.cpr.2022.102131 35180632 10.1016/j.cpr.2022.102131

[CR8] Brice, S., Rodgers, J., Ingham, B., Mason, D., Wilson, C., Freeston, M., Le Couteur, A., & Parr, J. R. (2021). The importance and availability of adjustments to improve access for autistic adults who need mental and physical healthcare: Findings from UK surveys. *British Medical Journal Open,**11*(3), e043336. 10.1136/bmjopen-2020-043336 10.1136/bmjopen-2020-043336 PMC7978247 33737429

[CR9] Brookman-Frazee, L., Drahota, A., Stadnick, N., & Palinkas, L. A. (2012). Therapist perspectives on community mental health services for children with autism spectrum disorders. *Administration and Policy in Mental Health and Mental Health Services Research,**39*(5), 365–373. 10.1007/s10488-011-0355-y 21533846 10.1007/s10488-011-0355-y PMC3546545

[CR10] Bury, S. M., Jellett, R., Spoor, J. R., & Hedley, D. (2020). “It defines Who I Am” or “It’s Something I Have”: What language do [autistic] Australian adults [on the autism spectrum] prefer? *Journal of Autism and Developmental Disorders*. 10.1007/s10803-020-04425-3 10.1007/s10803-020-04425-3 32112234

[CR11] Camm-Crosbie, L., Bradley, L., Shaw, R., Baron-Cohen, S., & Cassidy, S. (2019). ‘People like me don’t get support’: Autistic adults’ experiences of support and treatment for mental health difficulties, self-injury and suicidality. *Autism,**23*(6), 1431–1441. 10.1177/1362361318816053 30497279 10.1177/1362361318816053 PMC6625034

[CR12] Cassidy, S., Bradley, L., Shaw, R., & Baron-Cohen, S. (2018). Risk markers for suicidality in autistic adults. *Molecular Autism,**9*(1), 42. 10.1186/s13229-018-02264 30083306 10.1186/s13229-018-02264 PMC6069847

[CR13] *Cooper, K., Loades, M. E., & Russell, A. J. (2018). Adapting psychological therapies for autism–therapist experience, skills and confidence. *Research in Autism Spectrum Disorders,**45*, 43–50. 10.1016/j.rasd.2017.11.002 30245739 10.1016/j.rasd.2017.11.002 PMC6150418

[CR14] Crane, L., Adams, F., Harper, G., Welch, J., & Pellicano, E. (2019). ‘Something needs to change’: Mental health experiences of young autistic adults in England. *Autism,**23*(2), 477–493. 10.1177/1362361318757048 29415558 10.1177/1362361318757048

[CR54] Dickson, K. S., Lind, T., Jobin, A., Kinnear, M., Lok, H., & Brookman-Frazee, L. (2021). Correction to: A Systematic Review of Mental Health Interventions for ASD: Characterizing Interventions, Intervention Adaptations, and Implementation Outcomes. *Administration and Policy in Mental Health and Mental Health Services Research*, *48*(5), 884–908. 10.1007/s10488-021-01144-4 34196884 10.1007/s10488-021-01144-4 PMC12771532

[CR15] Duden, G. S. (2021). Challenges to qualitative evidence synthesis–aiming for diversity and abstracting without losing meaning. *Methods in Psychology,**5*, 100070. 10.1016/j.metip.2021.100070 10.1016/j.metip.2021.100070

[CR16] Fletcher-Watson, S., Adams, J., Brook, K., Charman, T., Crane, L., Cusack, J., Leekam, S., Milton, D., Parr, J. R., & Pellicano, E. (2019). Making the future together: Shaping autism research through meaningful participation. *Autism,**23*(4), 943–953. 10.1177/1362361318786721 30095277 10.1177/1362361318786721 PMC6512245

[CR17] Gillespie-Lynch, K., Kapp, S. K., Brooks, P. J., Pickens, J., & Schwartzman, B. (2017). Whose expertise is it? Evidence for autistic adults as critical autism experts. *Frontiers in Psychology*. 10.3389/fpsyg.2017.00438 28400742 10.3389/fpsyg.2017.00438 PMC5368186

[CR18] *Heijnen-Kohl, S. M. J., Hitzert, B., Schmidt, R., Geurts, H. M., & van Alphen, S. P. J. (2022). Features and needs of autistic older adults: A Delphi study of clinical experiences. *Clinical Gerontologist*. 10.1080/07317115.2022.2060157 35426768 10.1080/07317115.2022.2060157

[CR19] Hollocks, M. J., Lerh, J. W., Magiati, I., Meiser-Stedman, R., & Brugha, T. S. (2019). Anxiety and depression in adults with autism spectrum disorder: A systematic review and meta-analysis. *Psychological Medicine,**49*(4), 559–572. 10.1017/S0033291718002283 30178724 10.1017/S0033291718002283

[CR20] Horwood, J., Cooper, K., Harvey, H., Davies, L., & Russell, A. (2021). The experience of autistic adults accessing adapted cognitive behaviour therapy: ADEPT (autism depression trial) qualitative evaluation. *Research in Autism Spectrum Disorders,**86*, 101802. 10.1016/j.rasd.2021.101802 10.1016/j.rasd.2021.101802

[CR21] Hossain, M. M., Khan, N., Sultana, A., Ma, P., McKyer, E. L. J., Ahmed, H. U., & Purohit, N. (2020). Prevalence of comorbid psychiatric disorders among people with autism spectrum disorder: An umbrella review of systematic reviews and meta-analyses. *Psychiatry Research,**287*, 112922. 10.1016/j.psychres.2020.112922 32203749 10.1016/j.psychres.2020.112922

[CR22] Howlin, P. (2021). Adults with autism: Changes in understanding since DSM-111. *Journal of Autism and Developmental Disorders,**51*(12), 4291–4308. 10.1007/s10803-020-04847-z 33474661 10.1007/s10803-020-04847-z PMC8531125

[CR23] Howlin, P., & Magiati, I. (2017). Autism spectrum disorder: Outcomes in adulthood. *Current Opinion in Psychiatry,**30*(2), 69–76. 10.1097/YCO.0000000000000308 28067726 10.1097/YCO.0000000000000308

[CR24] *Hume, R. (2022). Show me the real you: enhanced expression of rogerian conditions in therapeutic relationship building with autistic adults. *Autism in Adulthood,**4*(2), 151–163. 36605974 10.1089/aut.2021.0065 PMC9645675

[CR25] Kenny, L., Hattersley, C., Molins, B., Buckley, C., Povey, C., & Pellicano, E. (2016). Which terms should be used to describe autism? Perspectives from the UK autism community. *Autism: the International Journal of Research and Practice,**20*(4), 442–462. 10.1177/1362361315588200 26134030 10.1177/1362361315588200

[CR26] *Kinnaird, E., Norton, C., & Tchanturia, K. (2017). Clinicians’ views on working with anorexia nervosa and autism spectrum disorder comorbidity: A qualitative study. *BMC Psychiatry,**17*(1), 292. 10.1186/s12888-017-1455-3 28797223 10.1186/s12888-017-1455-3 PMC5553805

[CR27] Kinnaird, E., Stewart, C., & Tchanturia, K. (2019). Investigating alexithymia in autism: A systematic review and meta-analysis. *European Psychiatry,**55*, 80–89. 10.1016/j.eurpsy.2018.09.004 30399531 10.1016/j.eurpsy.2018.09.004 PMC6331035

[CR28] Lai, M.-C., Kassee, C., Besney, R., Hull, L., Mandy, W., Szatmari, P., & Ameis, S. H. (2019). Prevalence of co-occurring mental health diagnoses in the autism population: A systematic review and meta-analysis. *The Lancet Psychiatry,**6*(10), 819–829. 10.1016/S2215-0366(19)30289-5 31447415 10.1016/S2215-0366(19)30289-5

[CR29] Lever, A. G., & Geurts, H. M. (2016). Psychiatric co-occurring symptoms and disorders in young, middle-aged, and older adults with autism spectrum disorder. *Journal of Autism and Developmental Disorders,**46*(6), 1916–1930. 10.1007/s10803-016-2722-8 26861713 10.1007/s10803-016-2722-8 PMC4860203

[CR30] Linden, A., Best, L., Elise, F., Roberts, D., Branagan, A., Tay, Y. B. E., Crane, L., Cusack, J., Davidson, B., Davidson, I., Hearst, C., Mandy, W., Rai, D., Smith, E., & Gurusamy, K. (2022). Benefits and harms of interventions to improve anxiety, depression, and other mental health outcomes for autistic people: A systematic review and network meta-analysis of randomised controlled trials. *Autism: the International Journal of Research and Practice*. 10.1177/13623613221117931 35957523 10.1177/13623613221117931 PMC9806485

[CR31] Lockwood, C., Munn, Z., & Porritt, K. (2015). Qualitative research synthesis: Methodological guidance for systematic reviewers utilizing meta-aggregation. *International Journal of Evidence-Based Healthcare,**13*(3), 179–187. 10.1097/XEB.0000000000000062 26262565 10.1097/XEB.0000000000000062

[CR32] *Maddox, B. B., Crabbe, S., Beidas, R. S., Brookman-Frazee, L., Cannuscio, C. C., Miller, J. S., Nicolaidis, C., & Mandell, D. S. (2020). “I Wouldn’t Know Where to Start”: Perspectives from clinicians, agency leaders, and autistic adults on improving community mental health services for autistic adults. *Autism the International Journal of Research and Practice,**24*(4), 919–930. 10.1177/1362361319882227 31674198 10.1177/1362361319882227 PMC7192780

[CR33] McGowan, J., Sampson, M., Salzwedel, D. M., Cogo, E., Foerster, V., & Lefebvre, C. (2016). PRESS peer review of electronic search strategies: 2015 guideline statement. *Journal of Clinical Epidemiology,**75*, 40–46. 10.1016/j.jclinepi.2016.01.021 27005575 10.1016/j.jclinepi.2016.01.021

[CR34] Milton, D. E. M. (2012). On the ontological status of autism: The ‘double empathy problem.’ *Disability & Society,**27*(6), 883–887. 10.1080/09687599.2012.710008 10.1080/09687599.2012.710008

[CR35] Mitchell, P., Sheppard, E., & Cassidy, S. (2021). Autism and the double empathy problem: Implications for development and mental health. *British Journal of Developmental Psychology,**39*(1), 1–18. 10.1111/bjdp.12350 33393101 10.1111/bjdp.12350

[CR36] *Mitran, C. (2022). Experiences of licensed counselors and other licensed mental health providers working with neurodiverse adults: An instrumental case study. *The Family Journal*. 10.1177/10664807221104138 10.1177/10664807221104138

[CR38] National Institute for Health and Care Excellence (NICE). (2021). *Autism spectrum disorder in adults: Diagnosis and management*. https://www.nice.org.uk/guidance/cg142 32186834

[CR39] Norcross, J. C. (2010). The therapeutic relationship. In B. L. Duncan, S. D. Miller, B. E. Wampold, & M. A. Hubble (Eds.), *The heart and soul of change: Delivering what works in therapy* (pp. 113–141). Washington: American Psychological Association. 10.1037/12075-004

[CR40] Page, M. J., McKenzie, J. E., Bossuyt, P. M., Boutron, I., Hoffmann, T. C., Mulrow, C. D., Shamseer, L., Tetzlaff, J. M., Akl, E. A., Brennan, S. E., Chou, R., Glanville, J., Grimshaw, J. M., Hróbjartsson, A., Lalu, M. M., Li, T., Loder, E. W., Mayo-Wilson, E., McDonald, S., & Moher, D. (2021). The PRISMA 2020 statement: An updated guideline for reporting systematic reviews. *Systematic Reviews,**10*(1), 89. 10.1186/s13643-021-01626-4 33781348 10.1186/s13643-021-01626-4 PMC8008539

[CR41] Pellicano, E., Fatima, U., Hall, G., Heyworth, M., Lawson, W., Lilley, R., Mahony, J., & Stears, M. (2022). A capabilities approach to understanding and supporting autistic adulthood. *Nature Reviews Psychology*. 10.1038/s44159-022-00099-z 36090460 10.1038/s44159-022-00099-z PMC9443657

[CR42] *Petty, S., Bergenheim, M.-L., Mahoney, G., & Chamberlain, L. (2021). Adapting services for autism: Recommendations from a specialist multidisciplinary perspective using freelisting. *Current Psychology*. 10.1007/s12144-021-02061-3 10.1007/s12144-021-02061-3

[CR43] *Petty, S., Donaldson, C., Whetton, J., & Baxter, N. (2022). Recommended adaptations to therapy services for autistic adults from specialist clinicians. *Current Psychology*. 10.1007/s12144-022-04034-6 10.1007/s12144-022-04034-6

[CR44] *Russell, A., Gaunt, D., Cooper, K., Horwood, J., Barton, S., Ensum, I., Ingham, B., Parr, J., Metcalfe, C., Rai, D., Kessler, D., & Wiles, N. (2019). Guided self-help for depression in autistic adults: The ADEPT feasibility RCT. *Health Technology Assessment,**23*(68), 1–94. 31856942 10.3310/hta23680 PMC6943380

[CR45] *Siddell, P. (2022). Psychologists’ views on the accessibility and effectiveness of psychological therapies for people with intellectual disabilities and autism. *Advances in Mental Health and Intellectual Disabilities,**16*(3), 147–156. 10.1108/AMHID-11-2021-0041 10.1108/AMHID-11-2021-0041

[CR46] Sizoo, B. B., & Kuiper, E. (2017). Cognitive behavioural therapy and mindfulness based stress reduction may be equally effective in reducing anxiety and depression in adults with autism spectrum disorders. *Research in Developmental Disabilities,**64*, 47–55. 10.1016/j.ridd.2017.03.004 28342404 10.1016/j.ridd.2017.03.004

[CR47] *Spain, D., Rumball, F., O’Neill, L., Sin, J., Prunty, J., & Happé, F. (2017). Conceptualizing and treating social anxiety in autism spectrum disorder: A focus group study with multidisciplinary professionals. *Journal of Applied Research in Intellectual Disabilities,**30*(S1), 10–21. 10.1111/jar.12320 28000357 10.1111/jar.12320

[CR48] The British Psychological Society. (2021). *Working with autism: Best practice guidelines for psychologists*. The British Psychological Society. https://www.bps.org.uk/psychologist/working-autism

[CR49] The Psychological Society of Ireland (2022). *Professional Practice Guidelines for the Assessment, Formulation, and Diagnosis of Autism in Children and Adolescents. The Psychological Society of Ireland.* https://www.psychologicalsociety.ie/source/PSI%20Autism%20Guidelines%202022%20(Interactive%20Version).pdf

[CR50] Thomas, J., & Harden, A. (2008). Methods for the thematic synthesis of qualitative research in systematic reviews. *BMC Medical Research Methodology,**8*(1), 45. 10.1186/1471-2288-8-45 18616818 10.1186/1471-2288-8-45 PMC2478656

[CR51] Tong, A., Flemming, K., McInnes, E., Oliver, S., & Craig, J. (2012). Enhancing transparency in reporting the synthesis of qualitative research: ENTREQ. *BMC Medical Research Methodology,**12*(1), 181. 10.1186/1471-2288-12-181 23185978 10.1186/1471-2288-12-181 PMC3552766

[CR56] Vivanti, G. (2019). Ask the Editor: What is the Most Appropriate Way to Talk About Individuals with a Diagnosis of Autism? *Journal of Autism and Developmental Disorders*, *50*, 691–693. 10.1007/s10803-019-04280-x 10.1007/s10803-019-04280-x 31676917

[CR52] White, S. W., Simmons, G. L., Gotham, K. O., Conner, C. M., Smith, I. C., Beck, K. B., & Mazefsky, C. A. (2018). Psychosocial treatments targeting anxiety and depression in adolescents and adults with autism spectrum disorder: Review of the latest research and recommended future directions. *Current Psychiatry Reports,**20*(10), 82. 10.1007/s11920-018-0949-0 30155584 10.1007/s11920-018-0949-0 PMC6421847

